# Video Head Impulse Test Findings in Patients With Benign Paroxysmal Positional Vertigo Secondary to Idiopathic Sudden Sensorineural Hearing Loss

**DOI:** 10.3389/fneur.2022.877777

**Published:** 2022-06-02

**Authors:** Yingzhao Liu, Yangming Leng, Renhong Zhou, Jingjing Liu, Hongchang Wang, Kaijun Xia, Bo Liu, Hongjun Xiao

**Affiliations:** Department of Otorhinolaryngology, Union Hospital, Tongji Medical College, Huazhong University of Science and Technology, Wuhan, China

**Keywords:** benign paroxysmal positional vertigo (BPPV), video head impulse test (vHIT), caloric test, idiopathic sudden sensorineural hearing loss (ISSNHL), vestibulo-ocular reflex (VOR)

## Abstract

Benign paroxysmal positional vertigo (BPPV) is amongst the most common causes of episodic vestibular syndrome. It can be classified as idiopathic and secondary types according to the causative factors, and the underlying mechanism between idiopathic (i-BPPV) and secondary BPPV (s-BPPV) may differ. Idiopathic sudden sensorineural hearing loss (ISSNHL) has been considered as a common inner ear disease that precipitates s-BPPV. Yet, few studies have addressed the functional impairment of the semicircular canal (SCC) system in patients with s-BPPV associated with ISSNHL. Our purpose was to explore the pathophysiological mechanism and investigate the clinical implications of video head impulse test (vHIT) in these patients. Here, the clinical and laboratory data of patients with BPPV secondary to ISSNHL, including the results of vHIT, were retrospectively reviewed, and compared with those of patients with i-BPPV. Pathological vHIT findings (low vestibulo-ocular reflex gain and re-fixation saccade), which mainly affected the posterior SCC, were more common in the s-BPPV group than in the i-BPPV group (41.9 and 0%, respectively). The incidence of horizontal SCC involvement was also higher in the s-BPPV group (45.16 and 16.67%, respectively). Furthermore, patients with s-BPPV showed lower vHIT gains of the posterior and horizontal SCCs in affected ears than in unaffected ears. Compared to i-BPPV, posterior SCC paresis detected by vHIT is more prevalent in BPPV secondary to ISSNHL. This dysfunction may be associated mainly with vestibular impairments caused by ISSNHL, and not with BPPV *per se*.

## Introduction

Sudden sensorineural hearing loss (SSNHL) is defined as a sensorineural hearing loss of 30 dB or more in at least three consecutive frequencies occurring within 72 hours (1). In most cases, no specific cause for the hearing loss can be identified, and these patients are classified as idiopathic SSNHL (ISSNHL). Numerous etiologies have been proposed, such as vascular insufficiency, viral infection, and immunologic reaction.

Clinically, about 30% of patients with ISSNHL also manifested vestibular symptoms, such as vertigo or imbalance (2). These vestibular symptoms can take the form of an acute vestibular syndrome (similar to vestibular neuritis, VN) (3), or an episodic vestibular syndrome, for instance, benign paroxysmal positional vertigo (BPPV) (4). BPPV is believed to be caused by detached otoconia from the utricular maculae, which migrate into the semicircular canals (SCCs) and may either move freely in the endolymph (canalithiasis) or become attached to the cupula (cupulolithiasis). Although its etiology is still elusive, BPPV falls into idiopathic and secondary categories according to causative factors, the latter include trauma, Ménière's disease, SSNHL, VN, etc. Recently, some studies have explored BPPV associated with ISSNHL, regarding to its possible pathophysiological mechanism, clinical characteristics, treatment outcomes, and prognosis (4, 5).

To examine the pathophysiological features of patients with vestibular impairments associated with SSNHL, many instrumental vestibular evaluations, such as caloric test and vestibular evoked myogenic potentials (VEMPs), have been explored (6, 7). Traditionally, the caloric test evaluates the vestibulo-ocular reflex (VOR) function of the horizontal SCC using non-physiological stimulus within the frequency range of 0.002–0.004 Hz (8). As a newly developed test, the video head impulse test (vHIT) assesses the angular VOR function of six SCCs within the physiological frequency range (5–7 Hz) (9). For ISSNHL patients with acute vertigo, some novel characteristics of vestibular lesions have been detected by vHIT recently (3, 10). Most of these patients exhibited posterior SCC dysfunction (3, 11), which could serve as a specific prognostic tool for predicting poor hearing recovery in patients with ISSNHL (12). VN, another typical variant of acute vestibular syndrome, shows mainly vestibular impairment in the horizontal and anterior SCC (as in superior VN). Infrequently, the posterior SCC may also be involved, which occurs in inferior or total VN (13–15). As for patients with BPPV secondary to ISSNHL, a common episodic vestibular syndrome following ISSNHL, few studies have so far addressed functional impairment in the SCC system (3, 16).

In this study, we retrospectively reviewed the clinical and laboratory data of patients with BPPV secondary to ISSNHL, including the results of vHIT. Our purpose was to investigate the clinical implications of vHIT in these patients.

## Materials and Methods

### Study Population

A single-center retrospective chart review was conducted at the Department of Otorhinolaryngology, Union Hospital affiliated to Tongji Medical College, Huazhong University of Science and Technology, Wuhan, China.

The clinical data of patients with BPPV secondary to ISSNHL who attended our outpatient clinic from 2016 January to 2021 October were retrospectively reviewed. Inclusion criteria were: (1) the diagnosis of ISSNHL is established according to the clinical practice guideline of sudden hearing loss proposed by the American Academy of Otolaryngology–Head and Neck Surgery (AAO-HNS) in 2012 (1); (2) BPPV is diagnosed according to the diagnostic criteria proposed by the Barany Society in 2015 (17), based on the Dix-Hallpike and Roll tests. (3) Patients underwent both the caloric test and vHIT. In addition, a series of consecutive patients with idiopathic BPPV (i-BPPV) were enrolled as control subjects. According to the diagnostic criteria (17), the included BPPV subtypes were: canalithiasis of the posterior canal (PC-BPPV), canalithiasis of the horizontal canal (HC-BPPV-CA), and cupulolithiasis of the horizontal canal (HC-BPPV-CU).

Exclusion criteria were: (1) other concurrent vestibular disorders (Ménière's disease, VN, vestibular migraine, etc.); (2) BPPV secondary to other disease, such as head trauma, VN, and Ménière's disease; (3) middle ear infections (otitis media, mastoiditis, etc.); (4) middle or inner ear anomaly; (5) having received previous ear surgery; (6) retrocochlear lesions; and (7) central nervous system disorders (multiple sclerosis, cerebellar infarction, etc.).

This study was conducted in strict accordance with the tenets of the Declaration of Helsinki. Informed consent was obtained from each patient, and the project was approved by the ethical committee of Union hospital, Tongji Medical College, Huazhong University of Science and Technology, Wuhan, China.

### Examination Procedure

The diagnosis of BPPV was established based on a history of recurrent positional vertigo and the presence of typical positional nystagmus on the Dix-Hallpike and roll test. For patients with secondary BPPV (s-BPPV), comprehensive neurotologic evaluations, including audiometry, videonystagmography, caloric test, and vHIT were conducted on the same day. Alternatively, patients with i-BPPV only received the vHIT. For all patients with ISSNHL, non-contrast routine magnetic resonance imaging (MRI) was performed to rule out retro-cochlear pathology. If a retro-cochlear lesion was suspected, contrast-enhanced MRI would be ordered.

#### Pure Tone Audiometry

After excluding middle ear pathologies by otoscopic examination and tympanometry test, a pure tone audiometry test was conducted in a sound-proof cabin in the frequency range of 0.25–8 kHz. Pure tone average (PTA) was calculated as the simple arithmetic mean for the frequencies of 0.25, 0.5, 1.0, 2.0, 4.0, and 8.0 kHz. Configurations of initial audiogram were categorized into four types (7): high-frequency hearing loss, low-frequency hearing loss, flat-type hearing loss, and profound hearing loss (a flat audiogram with a threshold shift >90 dB at all frequencies).

#### Video Head Impulse Test

The vHIT was conducted using an ICS Impulse system (GN Otometrics, Denmark) following the manufacturer's instructions by experienced technicians. Each patient wore a pair of lightweight, tight-fitting goggles equipped with a small video oculography camera to record and analyze the eye movement. Each patient was seated upright facing the wall 1.0 m away and was instructed to fixate a stationary target on the wall. The patient's head was passively and randomly rotated with a low amplitude (5–15°) and at a high peak velocity (150–250°/s) in an abrupt, brief, and unpredictable manner. At least 20 head impulses were delivered in each direction. Refixation saccades were categorized, against their appearance, as covert and overt. If the velocity of the saccade exceeded 50°/s, they were deemed positive. In the present study, it was considered abnormal if the horizontal vHIT gain <0.8 or vertical vHIT gain <0.7 and refixation saccades appeared.

#### Caloric Test

The bithermal caloric test was conducted using infrared videonystagmography (Visual Eyes VNG, Micromedical Technologies, Chatham, IL, USA). The subject was placed in a supine position with their head and upper trunk elevated at 30°. Each ear was alternately irrigated with a constant flow of air, with the temperature for warm and cool stimulation set at 50 and 24°C, respectively. The duration of each caloric irrigation lasted 60 s. Upon each irrigation, the maximum slow phase velocity (SPV_max_) of the caloric nystagmus was measured, and the canal paresis (CP) was calculated by using the Jongkees' formula. In this study, if the interaural asymmetry of the caloric nystagmus was ≥ 25%, CP was considered to be significant in the horizontal SCC, indicating an abnormal caloric response. According to the published criteria, if the summed SPVmax was <20°/s under four stimulation conditions, the caloric response is believed to indicate bilateral vestibular hypofunction. In this case, ice water irrigation (4°C, 1.0 ml) would be used to confirm the caloric unresponsiveness.

### Treatment and Follow-Up

Our treatment protocol conforms to the Chinese guideline for diagnosis and treatment of sudden deafness (2015) (18). Prednisolone was administered orally at a dose of 60 mg daily for six consecutive days, followed by a taper of 30, 20, 10, and 5 mg each for 2 days. Patients with lower body weight (<60 kg) were started on 1 mg/kg oral prednisolone daily, with this amount gradually tapered. Additional medication included vasoactive drugs (ginkgo biloba extract) and anticoagulant thrombolytic drugs (fibrinolytic enzyme). If conservative treatment failed, i.e., no hearing improvement after a 2-week conservative therapy, subsequent intratympanic dexamethasone injection (two times weekly for 2 weeks) or hyperbaric oxygen therapy would be suggested.

Canalith repositioning procedures (CRPs) were performed in both the s-BPPV and i-BPPV groups based on the subtypes of BPPV. Patients with PC-BPPV received the Epley procedure, while patients with HC-BPPV received the Gufoni maneuver (19). Patients were scheduled to return to the clinic at intervals of approximately 1 week until symptoms and typical positional nystagmus during the triggering maneuver completely disappeared.

According to the clinical practice guideline (AAO-HNS, 2012) (1), patients with a hearing gain of <15 dB (change in PTA, in decibels) were considered as treatment non-responders (NR), and the patients with a hearing gain ≥15 dB were classified as treatment responders. Treatment responders were further divided into three groups: (1) recovered to a hearing level within 10 dB of the unaffected ear (complete recovery, CR), (2) recovered to at least 50% of the maximum possible recovery (good recovery, GR), and (3) recovered below 50% of the maximum possible recovery (poor recovery, PR). Maximum possible recovery is defined as reaching the hearing level of the contralateral ear, which was considered as the baseline of normal hearing.

### Statistical Analyses

Statistical analysis was performed with SPSS software (version R26.0.0.2). All continuous variables are presented as means ± standard deviations (SD) or median and interquartile range (IQR 25th to 75th percentiles) after the verification of normal distribution. Quantitative data with normal distribution between subgroups were compared using the independent-sample *t*-test or paired *t-*test. Non-normally distributed data were compared using the Mann–Whitney *U* test or Wilcoxon signed-rank test. The Chi-squared test and Fisher exact test were performed for categorical variables. The values of *p* < 0.05 were assumed as statistically significant.

## Results

From 2016 to 2021, a total of 47 patients were diagnosed as BPPV secondary to ISSNHL, and 31 of them completed instrumental vestibular tests battery, including vHIT and caloric test. Additionally, 30 consecutive patients with i-BPPV were enrolled as a control group.

### Comparisons Between Patients With s-BPPV and Those With i-BPPV

The demographic and clinical characteristics of the patients with s-BPPV and i-BPPV are summarized in Table 1. No differences were observed in terms of gender (χ^2^ = 1.575, *p* = 0.21), age (*t* = −0.051, *p* = 0.959), and course duration (*U* = 448.5, *p* = 0.812) between the two groups.

**Table 1:** Demographic and clinical characteristics of patients with s-BPPV and i-BPPV.

	**s-BPPV (*n* = 31)**	**i-BPPV (*n* = 30)**
Gender (male/female)	13/18	8/22
Age (yr.)	53.03 ± 12.35	53.20 ± 13.21
Course duration (days)	12 (6, 43)	15.5 (6.5, 30)
Subtype of BPPV (PC-BPPV/HC-BPPV-CA/HC-BPPV-CU)	17/7/7	25/4/1

*s-BPPV, secondary benign paroxysmal positional vertigo; i-BPPV, idiopathic benign paroxysmal positional vertigo; PC-BPPV, canalithiasis of the posterior canal; HC-BPPV-CA, canalithiasis of the horizontal canal; HC-BPPV-CU, cupulolithiasis of the horizontal canal*.

**Figure 1:**
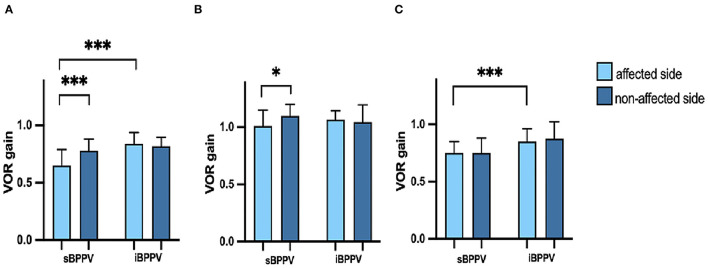
**(A)** Vestibular-ocular reflex (VOR) gains of the posterior semicircular canal (SCC) in patients with secondary benign paroxysmal positional vertigo (s-BPPV) and idiopathic BPPV (i-BPPV); **(B)** VOR gains of horizontal SCC in patients with s-BPPV and i-BPPV; **(C)** VOR gains of the anterior SCC in patients with s-BPPV and i-BPPV. An upper end of the box is the median. The whisker is the interquartile range (IQR). *p < 0.05; ***p < 0.001.

In the s-BPPV group, 13 cases (13/31, 41.9%) showed pathological vHIT findings (low VOR gain and refixation saccade), with the posterior SCC being the most affected (13/13), followed by the horizontal SCC (5/13). Meanwhile, all patients with i-BPPV showed normal vHIT results. Compared to their idiopathic counterparts, patients with s-BPPV had lower vHIT gains in the posterior (*t* = −5.280, *p* < 0.001) and anterior SCC (*t* = −4.575, *p* < 0.001) of the affected side. No significant difference was demonstrated between the two groups regarding vHIT gain in the horizontal SCC (*t* = −1.320, *p* = 0.192) (Figure 1).

The mean VOR gains for the anterior, horizontal, and posterior SCC of both affected and non-affected ears are shown in Table 2. In the s-BPPV group, VOR gains of the horizontal (*t* = −2.566, *p* = 0.016) and posterior SCC (*t* = −5.673, *p* < 0.001) in the affected ears were lower than those in the unaffected ears, respectively (Figure 1). No interaural difference for VOR gains of the anterior SCC was observed in either group (Figure 1).

**Table 2:** vHIT gains of the anterior, horizontal, and posterior semicircular canals in patients with s-BPPV and i-BPPV.

**SCCs**		**vHIT gains**	
		**s-BPPV**	**i-BPPV**
Anterior SCC	Affected side	0.73 ± 0.14	0.88 ± 0.11
	Non-affected side	0.75 (0.70, 0.88)	0.89 ± 0.18
Horizontal SCC	Affected side	1.01 ± 0.21	1.07 ± 0.12
	Non-affected side	1.10 ± 0.12	1.07 ± 0.20
Posterior SCC	Affected side	0.61 ± 0.20	0.84 ± 0.12
	Non-affected side	0.78 (0.72, 0.88)	0.83 ± 0.11

*s-BPPV, secondary benign paroxysmal positional vertigo; i-BPPV, idiopathic benign paroxysmal positional vertigo; SCC, semicircular canal; vHIT, video head impulse test*.

**Figure 2:**
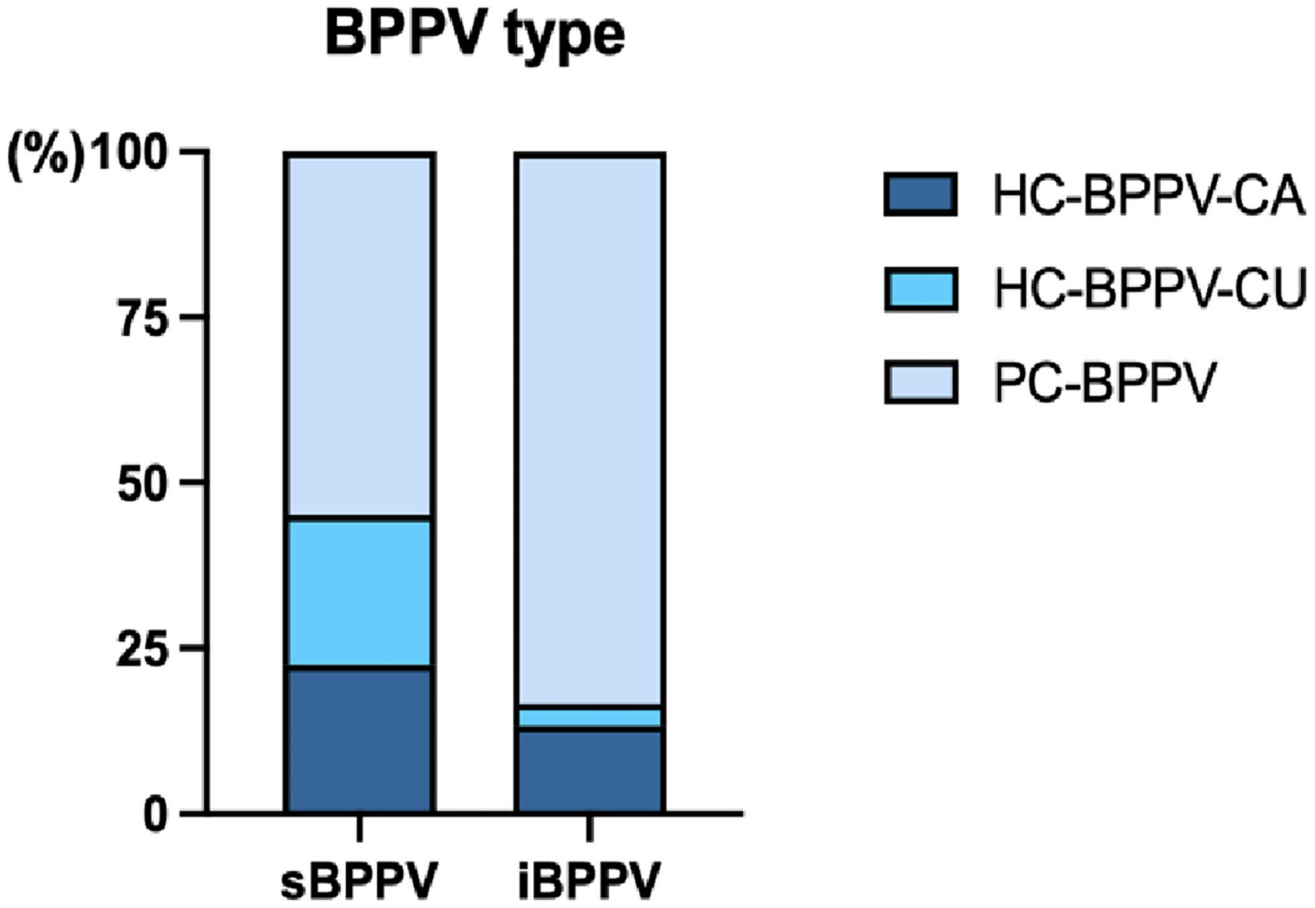
The proportion of posterior semicircular canal canalithiasis (PC-BPPV), horizontal semicircular canal canalithiasis (HC-BPPV-CA), or cupulolithiasis (HC-BPPV-CU) in patients with idiopathic BPPV (*n* = 30) and BPPV secondary to ISSNHL (*n* = 31).

There were 17 PC-BPPV (17/31, 54.84%), seven HC-BPPV-CA (7/31, 22.58%), and seven HC-BPPV-CU cases (7/31, 22.58%) in the s-BPPV group. And, the i-BPPV group consisted of 25 patients with PC-BPPV (25/30, 83.33%), four patients with HC-BPPV-CA (4/30, 13.33%), and only one patient with HC-BPPV-CU (1/30, 3.33%) (Table 1; Figure 2). The proportions of BPPV subtypes was significantly different between the two groups (χ^2^ = 7.407, *p* = 0.026). Pairwise comparison revealed that the proportion of PC-BPPV in the s-BPPV group was lower than that in the i-BPPV group. And, the HC-BPPV-CU was more prevalent in the s-BPPV group than in the i-BPPV group. If HC-BPPV-CA and HC-BPPV-CU were collectively classified as HC-BPPV, horizontal SCC was more susceptible to BPPV pathology in the s-BPPV group (14/31, 45.16%) than in the i-BPPV group (5/30, 16.67%) (χ^2^ = 5.772, *p* < 0.05).

In our s-BPPV group, most patients (27/31) suffered an acute vertigo attack simultaneously with a sudden hearing loss, lasting about 1–3 days. As acute vestibular symptoms gradually subsided, episodic positional vertigo became evident (Table 3). The remaining four patients with s-BPPV had no concomitant vestibular symptoms at the time of sudden hearing loss. Positional vertigo developed within 4 days after the onset of hearing loss. The median interval between the onset of postural vertigo and the diagnostic positional tests for patients with s-BPPV and those with i-BPPV was 12 and 15.5 days, respectively (Table 1).

**Table 3:** Demographic and clinical characteristics of patients with s-BPPV with abnormal vHIT results and those with normal vHIT results.

		**Abnormal vHIT (*n* = 13)**	**Normal vHIT (*n* = 18)**	**Test statistics**	***P*-value**
Gender (male/female)		5/8	8/10	χ^2^ = 0.111	0.739
Age (yr.)		52.46 ± 13.59	53.44 ± 11.77	*t* = −0.215	0.831
Course duration (days)		8 (3.5, 57.5)	14 (6, 33.25)	U = 106.5	0.674
Accompanied symptom (with/without vertigo)		13/0	14/4	-	0.120
Audiogram configurations (up/down/flat/profound)		0/8/0/5	1/4/3/10	-	0.099
Outcome of hearing (CR/GR/PR/NR)		0/0/3/10	0/4/5/9	-	0.183
Rate of SN		53.85% (7/13)	16.67% (3/18)	-	0.052
Caloric test	CP value	53.85 ± 22.74	16.89 ± 12.17	*t* = 5.854	<0.001
	Abnormal rate	92.31% (12/13)	27.78% (5/18)	-	0.001
Type of BPPV (PC-BPPV/HC-BPPV-CA/HC-BPPV-CU)		5/3/5	12/4/2	χ^2^ = 3.487	0.168
vHIT gains of affected side	Anterior SCC	0.70 ± 0.16	0.75 ± 0.12	*t* = −1.066	0.295
	Horizontal SCC	0.86 ± 0.20	1.13 ± 0.14	*t* = −4.432	<0.001
	Posterior SCC	0.44 ± 0.17	0.74 ± 0.12	*t* = −5.752	<0.001

*SCC, semicircular canal; vHIT, video head impulse test; CR, complete recovery; GR, good recovery; PR, poor recovery; NR, non-responder; SN, spontaneous nystagmus; CP, canal paresis. Up-sloping and down-sloping audiogram configurations correspond to low-frequency and high-frequency hearing loss, respectively*.

### Comparison Between s-BPPV Patients With Normal vHIT and Those With Abnormal vHIT

There were 13 patients with abnormal vHIT and 18 patients with normal vHIT in the s-BPPV group. The demographic and clinical characteristics of these two subgroups are illustrated in Table 3. Age (χ^2^ = 0.111, *p* = 0.739), gender (*t* = −0.215, *p* = 0.831), and course duration (*U* = 106.5, *p* = 0.674) did not differ between patients with abnormal and normal vHIT findings.

No differences were observed between these two subgroups in the incidence of acute vertigo (*p* = 0.120), audiogram configurations (*p* = 0.099), hearing outcome (*p* = 0.183), and incidence of spontaneous nystagmus (*p* = 0.052).

Compared to patients with normal vHIT, those with abnormal vHIT showed a significantly increased CP value in the caloric test (*t* = 5.854, *p* < 0.001). Patients with abnormal vHIT were more likely to have an abnormal caloric response (*p* < 0.001).

**Figure 3:**
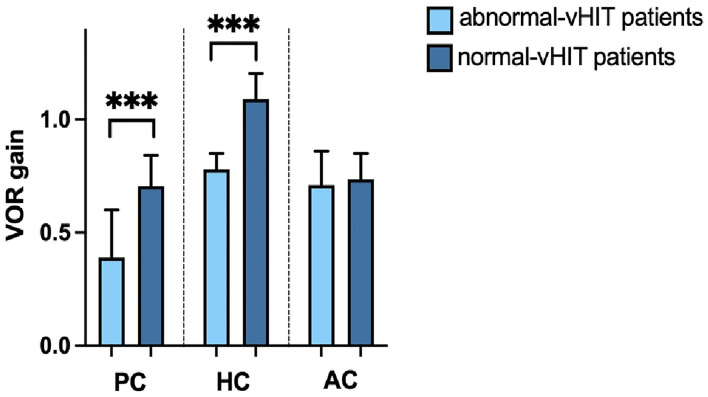
vHIT gains for the posterior canal (PC), horizontal canal (HC), and anterior canal (AC) of patients with s-BPPV with abnormal vHIT results and those with normal vHIT results. ***p < 0.001.

The VOR gains of the horizontal SCC (*t* = −4.432, *p* < 0.001) and posterior SCC (*t* = −5.752, *p* < 0.001) in the affected ears were significantly lower in the abnormal vHIT group than in the normal vHIT group, respectively. VOR gains of the anterior SCC in affected ears did not differ between the two subgroups (*t* = −1.066, *p* = 0.295) (Figure 3).

All patients were given a CRP according to BPPV subtypes. Three patients in the s-BPPV group and five patients in the i-BPPV group adhered to regular follow-up visits on weekly basis, and others were lost to follow-up. In two of the three s-BPPV patients, three CRP sessions were needed to completely resolve the positional vertigo and nystagmus, and in one of the five patients with i-BPPV, two CRP sessions were needed. Due to inadequate data, the number of CRPs in the two groups was not compared in this study.

## Discussion

### Comparisons Between Patients With s-BPPV and Those With i-BPPV

In this study, pathological vHIT response was common in the s-BPPV group, and the posterior and horizontal vHIT gains were lower in the affected side than those in the contralateral side. However, all patients in the i-BPPV group had normal vHIT, and VOR gains in the corresponding SCCs did not differ between the affected and non-affected sides. These results indicated that the pathological vHIT response in BPPV secondary to ISSNHL were mainly attributed to the vestibular impairments caused by ISSNHL, not BPPV *per se*.

In 2004, Rambold et al. for the first time, assessed the high-frequency VOR function in two patients with horizontal BPPV and ipsilateral hearing loss by using the three-dimensional scleral search coil technique. They reported vHIT gain deficit in the posterior and horizontal SCCs ipsilateral to the hearing loss ear (16). In a recent meta-analysis, by comparing the VOR gains of the three SCCs on the affected side relative to those in the contralateral side, and/or healthy controls, Elsherif et al. (20) detected a significant reduction of the posterior vHIT gain in patients with BPPV. Several explanations have been proposed. Disturbance of endolymphatic hydrodynamics in the posterior SCC due to free-floating particles (canalithiasis) has been suggested (21), but this hypothesis remains controversial as other investigators found no significant impairment of VOR gain for the affected posterior SCC in patients with PC-BPPV involving the ampullary arm (22, 23). Canalith jam, either partial or complete type, could lead to decreased VOR gain in response to vHIT. The mechanical occlusion of the narrow portion of SCC and resultant blockage of the endolymphatic flow may temporarily inhibit dynamic responses of cupular receptor to high-frequency stimulation (24). The jam also could produce a persistent transcupular pressure gradient and cause cupula deflection in either excitatory or inhibitory direction (25, 26). This cupular deflection may bias the optimal operating point of vestibular hair cells, thus decrease the cupular sensitivity to high-frequency stimulation, resulting in reduced VOR gain for vHIT (27, 28). In addition, a neuropathic mechanism of BPPV should be considered. A quantitative temporal bone study revealed significant neurodegeneration (over 50% of cell loss) in both the superior and inferior vestibular ganglion of patients with documented BPPV (29, 30). However, the etiology of BPPV, i.e., idiopathic or secondary, has not been specified in most of the included studies in this meta-analysis (21, 31–34). Our study showed that, compared with their idiopathic counterparts, patients with s-BPPV showed an obvious interaural difference in vHIT gains in the corresponding SCCs, which indicated that the underlying causes (idiopathic or secondary to ISSNHL) of BPPV may significantly impact the high-frequency VOR function.

We found that the abnormal vHIT response occurred most frequently in the posterior SCC, followed by the horizontal and anterior SCC. These findings were in line with previous studies, which have demonstrated that the reduced vHIT gain was most prevalent in the posterior SCC in the ISSNHL patients with acute vertigo (3, 10, 35). Anatomically, the shared susceptibility of posterior SCC and cochlea (and possibly saccule) may likely reflect the common vascular supply of the pars inferior of the labyrinth given by the common cochlear artery (36). On the other hand, inferior vestibular nerve innervates posterior SCC and saccular macula. Therefore, the lesion pattern might provide clues on a neural vs. vascular etiology in an acute setting. It has been suggested that posterior SCC paresis associated with ipsilateral sudden hearing loss could possibly be caused by ischemia of the common cochlear artery (37), while the lesion pattern of hearing loss and ipsilateral paresis of the posterior and horizontal SCCs may indicate viral neurolabyrinthitis (16). Recently, a selective vHIT gain reduction of the ipsilateral posterior SCC in a SSNHL patient with acute vertigo has been reported, in which posterior SCC fibrosis has been detected by inner ear MRI, thus lend further support to vascular etiology (38). Although recent application of instrumental testing (vHIT and VEMP) can facilitate topographical diagnosis of selective dysfunction of inner ear end-organs, the underlying mechanisms require further investigations for patients with vertigo in SSNHL (3, 38).

In our series, BPPV involving posterior SCC predominated in patients with i-BPPV (83.3%, 25/30). Meanwhile, posterior (PC-BPPV) and horizontal SCC (HC-BPPV-CA and HC-BPPV-CU) were almost equally affected in patients with s-BPPV [54.8% (17/31) and 45.2% (14/31), respectively]. Our findings were in agreement with those of Hong and Yeo, who reported that the most commonly involved canal was horizontal SCC in patients with both ISSNHL and BPPV (4). Additionally, multi-canal involvement was not uncommon in patients with BPPV secondary to ISSNHL (6, 39). In our series, no multiple SCCs involvement was identified, probably due to the relatively small sample size.

Notably, in the s-BPPV group, there is an inconsistency between SCC involvement by BPPV pathology and abnormal vHIT findings, that is, BPPV predominantly affected horizontal SCC while the pathological vHIT response usually occurred in posterior SCC. Conversely, VN patients are more likely to develop s-BPPV involving posterior SCC (40, 41) and to have pathological vHIT findings in horizontal SCC (13). It is suggested that VN could directly damage the utricular macula or disrupt nerve afferentiation (mainly in superior vestibular nerve), leading to otoconia detachment, but relatively spare the posterior SCC and inferior vestibular nerve function (42). Further in-depth study was warranted to explore the pathophysiological mechanisms underlying this BPPV pathology-vHIT inconsistency in patients with BPPV secondary to ISSNHL.

### Comparisons Between s-BPPV Patients With Normal vHIT and Those With Abnormal vHIT

No significant differences in the audiogram configurations and hearing outcomes were observed between patients with normal and those with abnormal vHIT results. In contrast, Byun et al. (12) found that abnormal vHIT gain in the posterior SCC appears to be a specific prognostic factor for unfavorable hearing recovery in ISSNHL. This discrepancy may be ascribed to the different inclusion criteria and smaller sample size of our study.

Compared with the caloric test, vHIT is a low-sensitivity, high-specificity test for detecting horizontal VOR pathology. It has been reported that horizontal canal vHIT is typically normal until a unilateral weakness score on caloric test of >62.5% is reached (43). In a series of patients complaining of vertigo or dizziness in a community hospital, Mahringer and Rambold (44) found that a pathological vHIT was dependent on the severity of unilateral weakness on caloric examination. We also demonstrated a significantly higher incidence of caloric weakness and CP value in patients with s-BPPV with abnormal vHIT results, which indicated more serious vestibular impairments in this subgroup. Furthermore, of the 13 vHIT- positive cases, 5 (38.5%) had abnormal horizontal vHIT results, while 12 (92.3%) had an abnormal caloric response. This horizontal vHIT-caloric dissociation may be attributed to vestibular compensation. Rapid angular head movements are frequent in daily activities. Considering a median 12-day clinical course, the response to rapid head movements may be better adapted than to non-physiological caloric stimuli (8).

In this s-BPPV series, the incidence of posterior and horizontal SCC involvement by BPPV pathology was not different between patients with normal and abnormal vHIT. This result may indicate that, for patients with BPPV secondary to ISSNHL, the severity of high-frequency VOR impairment may not be related to the involvement of SCCs. The relationship between the severity of vestibular damage and SCC involvement in BPPV secondary to ISSNHL needs to be further studied, as the small sample size may limit the generalizability of our findings.

This study is subject to the following limitations. Firstly, due to the small sample size, some uncommon subtypes of BPPV, such as multi-canal BPPV, were not identified in this series. Additionally, confounding factors were not investigated, as the number of patients with an abnormal vHIT response and those with a normal vHIT response in s-BPPV was small, although univariate analysis revealed no baseline differences between the two subgroups. Secondly, the horizontal apogeotropic positional nystagmus might be caused by cupulopathy of the horizontal SCC due to other etiologies, such as biochemical alteration of the inner ear fluids during the course of SSNHL or blood debris attaching to the cupula due to inner ear hemorrhage (37, 45, 46), because many patients were not followed up regularly as suggested by their physicians and the nystagmus conversion might be missed. Moreover, the treatment outcomes of CRP could not be statistically analyzed due to incomplete follow-up data. Several studies have shown that patients with s-BPPV need more CRPs for complete recovery than their idiopathic counterparts (6, 39). Therefore, the impact of pathological vHIT on the treatment outcomes of CRP in patients with s-BPPV warrants further investigations with large samples and long-term follow-up.

## Conclusions

Compared to i-BPPV, posterior SCC dysfunction detected by vHIT is more prevalent in BPPV secondary to ISSNHL. This dysfunction is mainly associated with vestibular impairments caused by ISSNHL rather than BPPV *per se*.

## Data Availability Statement

The original contributions presented in the study are included in the article/supplementary material, further inquiries can be directed to the corresponding authors.

## Ethics Statement

The studies involving human participants were reviewed and approved by the Ethical Committee of Union Hospital, Tongji Medical College, Huazhong University of Science and Technology, Wuhan, China. The patients/participants provided their written informed consent to participate in this study.

## Author Contributions

Material preparation and data collection were performed by YLi, RZ, JL, and HW. Data analysis was performed by YLi and KX. The first draft of this manuscript was written by YLe and BL. A critical review of this manuscript was performed by YLe, HX, and BL. All authors contributed to the study conception and design, read, and approved the final manuscript.

## Funding

This work was supported by the National Natural Science Foundation of China (NSFC No. 81670930), Natural Science Foundation of Hubei Province, China (No. 2021CFB547), and Fundamental Research Funds for the Central Universities, China (No. 2016YXMS240).

## Conflict of Interest

The authors declare that the research was conducted in the absence of any commercial or financial relationships that could be construed as a potential conflict of interest.

## Publisher's Note

All claims expressed in this article are solely those of the authors and do not necessarily represent those of their affiliated organizations, or those of the publisher, the editors and the reviewers. Any product that may be evaluated in this article, or claim that may be made by its manufacturer, is not guaranteed or endorsed by the publisher.
